# Ultrasound examination of the uterine diameter and the uterine blood vessels size in temporarily confined sows in the postpartal period– a pilot study

**DOI:** 10.1186/s40813-025-00448-3

**Published:** 2025-06-11

**Authors:** Tip-apa Akkhaphan, Preechaphon Taechamaeteekul, Alexander Grahofer, Padet Tummaruk

**Affiliations:** 1 Center of Excellence in Swine Reproduction, Department of Obstetrics, Gynaecology and Reproduction, Faculty of Veterinary Science, Chulalongkorn University, Bangkok, 10330 Thailand; 2 Clinic for Swine, Department for Clinical Veterinary Medicine, Vetsuisse Faculty, University of Bern, Bern, Switzerland

**Keywords:** Prolonged farrowing, Farrowing duration, Parturition, Reproduction, Ultrasound, Uterus

## Abstract

**Background:**

Prolonged farrowing duration is currently one of the challenges in the swine industry, which leads to postpartum complications by increasing uterine inflammation, and interference with physiological processes after parturition. The aim of the present study was to assess the relationship between uterine involution and uterine vessel size with farrowing duration in temporarily confined sows (during the day of farrowing and for the following 3 days) under tropical climates.

**Results:**

Fourteen Landrace × Yorkshire sows were included in the study. Uterine diameter and uterine vessel size were measured from 2 to 15 days postpartum using B-mode ultrasonography, a non-invasive technique that enables continuous monitoring of uterine involution and detection of reproductive problems in the same sows throughout the 15-day postpartum period. To determine the relationship between farrowing duration and uterine diameter, sows were categorized into two groups: sows with a farrowing duration of ≤ 300 min (normal) and sows with a farrowing duration > 300 min (prolonged). The mean farrowing duration was 200 ± 128 min, with 28.6% of sows experiencing prolonged farrowing. In the prolonged farrowing group, the diameter decreased from 43.5 ± 9.4 mm to 12.4 ± 0.5 mm, while in the normal group, it decreased from 30.7 ± 1.4 mm to 13.6 ± 0.4 mm. On day 2, sows in the prolonged farrowing group exhibited a higher uterine diameter than the normal group (*P* = 0.042). There was no significant difference in the size of the uterine blood vessels between the two groups (prolonged farrowing: 7.3 ± 1.3 mm to 2.5 ± 0.1 mm; normal farrowing 6.1 ± 0.7 mm to 2.7 ± 0.1 mm) (*P* = 0.397). However, the uterine diameter was correlated with the size of the uterine blood vessels in sows with either a normal farrowing duration (*r* = 0.705, *P* < 0.001) or a prolonged farrowing duration (*r* = 0.749, *P* < 0.001).

**Conclusions:**

This is the first study to evaluate and provide initial data on uterine vessel size and its correlation with the uterine diameter in postpartum sows. Although no significant differences between uterine vessel size and farrowing duration were detected, a notable increase in uterine size during the initial 2 days postpartum in temporarily confined sows under tropical climates with prolonged farrowing duration was identified.

## Background

In the last decade, the exponential growth of genetic development in the litter size of sows has had a significant impact on the swine industry, spreading worldwide. Several studies have been conducted regarding the impact of large litter size. The problems that arise when litter size is increased include decreased birth weight of piglets, increased incidence of farrowing assistance [1, 2], increased rate of intrauterine growth retardation (IUGR) [3, 4], impacting piglet immunity [5], decreased activity, and the need for more time for the first suckle [6]. Additionally, decreased colostrum intake occurs when the number of piglets exceeds the number of functional teats [6, 7]. One of the significant problems accompanying the increase in litter size is prolonged farrowing duration [5, 8].

In the modern swine industry, hyperprolific sows typically produce a total number of piglets born per litter of ≥ 16 [9, 10], and farrowing durations exceeding 300 min are generally considered prolonged [11]. A previous study conducted under tropical conditions revealed an average farrowing duration of 221 min, with 21.7% of sows experiencing prolonged farrowing duration [8]. In sows with prolonged farrowing duration, signs such as restlessness and intense abdominal straining due to acute obstructive dystocia were observed [12, 13]. Additionally, sows that were obese or constipated before farrowing should be carefully monitored, as constipation can lead to partial occlusion of the birth canal [14, 15].

Prolonged farrowing duration impacts both sow and piglets. A study demonstrated that the number of stillborn piglets in sows with long duration was higher than in those with short farrowing duration [16]. Furthermore, prolonged farrowing duration can lead to secondary uterine inertia, increasing the risk of dystocia [12, 13]. Another study showed that postpartum fever and loss of appetite are associated with an increased duration of farrowing [17]. Prolonged farrowing duration also suggests associations with reduced colostrum yields, placenta expulsion before the last piglet, retained placenta, increased amount of vaginal discharge, a higher prevalence of postpartum dysgalactia syndrome, impaired uterine involution, and reduced fertility [18–20].

During the postpartum period, the uterus returns to its nongravid size and restores itself to prepare for the next pregnancy [21], a process typically completed within 4 weeks postpartum [22, 23]. Normally, parturition initiates an inflammatory process, and if this process is prolonged, it can increase uterine inflammation, leading to delayed uterine involution and affecting reproductive performance in sows [24]. Moreover, monitored uterine involution is a tool that can investigate reproductive problems, and ultrasound is a suitable method to monitor postpartum diseases and measure uterine diameter in sows during the postpartum period [22, 23, 25, 26]. Real-time (B-mode) ultrasonography is a non-invasive technique that allows continuous measurement of uterine diameter in the same sows from the early postpartum period until completion of the uterine involution process, typically by 15 days postpartum [22, 23]. Initially, real-time (B-mode) ultrasonography was used in the swine industry for pregnancy detection. It has since been extended for the assessment of peri-pubertal and mature non-pregnant female sows due to its ease of access, safety, and time-saving advantages during examinations [27]. The increasing use of ultrasound to monitor the puerperal uterus and uterine involution has led to new studies.

A recent study demonstrated that rapid uterine involution occurs within the first week after parturition, leading to a significant reduction in uterine size, with the mean diameter decreasing from 32.5 mm on day 2 postpartum to 11.4 mm on day 12, representing a relative regression of 66% [28]. The rate of uterine involution is associated with body condition score, gestation length, and fever on the first day postpartum [28]. Delayed uterine involution can have a negative impact on sow fertility and cause substantial economic loss. Similarly, another study demonstrated that uterine height decreased from 11 cm in the first week to 5.9 cm in the last week, and uterine horn diameter decreased from 2.6 to 1.4 cm during the first week of lactation (days 1–7) [29]. Uterine height and uterine horn diameter decreased significantly faster during the first week (days 1–7) compared to the last week (days 22–28) [29]. Additionally, in human medicine, ultrasound is used to measure the uterine vessels. There was a study that investigated the average diameter of the right and left uterine arteries in humans, which was found to be 1.4 mm in 12 non-pregnant women [30]. Moreover, the average uterine artery diameter increases from 3 mm at the beginning of pregnancy to 7 mm at term [31]. The increase in uterine artery diameter during pregnancy reflects the elevated blood flow necessary to support fetal development and placental function. Postpartum, this parameter becomes particularly relevant as it can serve as an indirect marker of uterine involution, the physiological process by which the uterus returns to its pre-pregnant size and function. During involution, uterine blood flow decreases, accompanied by a gradual reduction in uterine artery diameter. Thus, monitoring changes in uterine vessel size through imaging techniques such as ultrasonography may offer valuable insights into the progression and completeness of the involution process. Delayed or incomplete uterine involution is associated with postpartum complications, including uterine infections and hemorrhage, making the assessment of uterine vascular dynamics a useful tool for evaluating postpartum reproductive health. To our knowledge, this aspect has not yet been investigated in pigs. In the modern swine industry, one of the major challenges associated with highly prolific sows is prolonged parturition, typically defined as a farrowing duration exceeding 300 min. Prolonged parturition is known to impair the farrowing process, increase the risk of retained placenta, and may potentially delay uterine involution [24]. However, the effects of extended farrowing duration on the overall process of uterine involution and the associated reduction in uterine vessel diameter in sows have not been thoroughly studied. Consequently, assessing the relationship between uterine vessel diameter and farrowing duration in sows is of significant interest. Understanding the underlying causes of these issues and exploring appropriate interventions is essential for minimizing their impact on postpartum recovery and reproductive performance. The present study aimed to examine the relationship between farrowing duration and uterine involution, defined as the post-parturition reduction in uterine diameter and blood vessel size, in sows housed in a temporarily confined system under tropical conditions.

## Materials and methods

### Study design

This study enrolled 14 Landrace × Yorkshire crossbred sows housed in a temporarily confined system in northern Thailand. The sows were distributed by parity as follows: parity 1 (*n* = 4), parity 2–3 (*n* = 5), and parity 4–6 (*n* = 5). All sow and piglet parameters were recorded, including sow identity, parity number, gestation length, body condition score, fecal score, backfat thickness, farrowing duration, the total number of piglets born per litter, the number of piglets born alive per litter, the number of mummified fetuses, stillborn piglets per litter, litter birth weight (kg), fever, vaginal discharges, and mastitis. Uterine involution was measured from day 2 to day 14 using ultrasound. To determine the relationship between farrowing duration, uterine diameter, and uterine vessel diameter, the sows were categorized into two groups: those with a farrowing duration of ≤ 300 min (normal, *n* = 10) and those > 300 min (prolonged, *n* = 4). All data were compared to identify relationships between each factor using statistical analysis. The sample size in the present study was estimated based on an expected mean difference in farrowing duration of 200 min between groups, with a pooled standard deviation of 40 min. To detect this difference with a significance level (α) of 0.05 and a statistical power of 0.80, the required sample size was approximately 2.1 sows per group, corresponding to a very large effect size (Cohen’s d = 5.0). Therefore, including 3–4 sows per group would be sufficient to detect a difference of this magnitude.

## Housing, and general management

The sows were housed in a closed environment equipped with an evaporative cooling system to regulate temperature and humidity. During the experimental timeframe, the barn’s interior maintained an average daily temperature of 27.4 ± 0.9 °C, fluctuating between a low of 25.8 °C and a high of 28.8 °C. Similarly, the average daily humidity level within the barn was recorded at 83.6 ± 5.2%, ranging from a minimum of 75% to a maximum of 94%. All sows were artificially inseminated and placed in a group housing system from 3 days after insemination until reaching 109 ± 2.0 days of gestation. Throughout the gestation period, sows received a daily feed allowance ranging from 3.0 to 3.5 kg, depending on their specific gestation phase. The gestation diet comprised 12.7% crude protein, 2700 kcal/kg of metabolizable energy, 5.7% fiber, and 0.7% lysine. Approximately one week before parturition, the sows were transferred to the farrowing unit. The farrowing pen was designed as a free-farrowing system and featured an adjustable swing hinge and a plastic-slatted floor. Each farrowing pen had dimensions of 2.00 × 2.35 × 0.90 m, providing a total space of 4.7 m² per pen. During the day of farrowing and for the following 3 days, the metal swing hinge was closed, and the sows were confined to individual crates measuring 1.80 × 0.60 × 0.90 m, allowing for a space of 1.08 m² per sow. The swing hinge was fully opened starting from 4 days postpartum until the weaning period. The creep area was equipped with a heating lamp, a rubber mattress, and a feeding bowl. Seven days before parturition, the feed was adjusted to a lactation diet, ranging from 3.0 to 3.5 kg/day. The lactation diet contained 17.2% crude protein, 3300 kcal/kg of metabolizable energy, 4.3% fiber, and 1.1% lysine. Feed, in pellet form, was provided four times daily at 06:00 a.m., 10:00 a.m., 01:00 p.m., and 04:00 p.m. After farrowing, sows were fed using an automatic feeding machine with an average daily feed intake of 5.0 to 6.0 kg per sow. Water was provided *ad libitum* through drinking nipples. The flow rate of water from drinking nipples for lactating sows was 1.5–2.0 L per minute. The farrowing process was supervised by experienced stock personnel with minimal disturbance. All sows were allowed to farrow naturally, and no manual extraction of piglets was performed in any of the sows included in the present study. After the birth of the 10th piglet, all sows received 10 IU of oxytocin (Phenix Pharmaceuticals N.V. Co. Ltd., Hoogstraten, Belgium) to aid in placental expulsion and milk let-down. Following farrowing, the rectal temperature of each sow was measured once daily in the morning. If a sow’s rectal temperature exceeded 40.0 °C on the first postpartum day or surpassed 39.5 °C on subsequent days, an antipyretic (3.0 mg/kg ketoprofen, Ketoprofen^®^, KELA N.V., Hoogstraten, Belgium) was administered. Additionally, all sows received an intramuscular injection of amoxicillin (10 mg/kg, Vetrimoxin L.A.^®^, Ceva Santé Animale, Libourne, France) for three consecutive days postpartum. The health status of the sows was monitored by a veterinarian. All sows were vaccinated against foot-and-mouth disease (AFTOPOR^®^, Merial SAS, Lyon, France), Aujeszky’s disease virus (Porcilis^®^ AD Begonia, Merck Animal Health, Madison, USA), classical swine fever (Ceva-Phylaxia Veterinary Biologicals Co. Ltd., Budapest, Hungary), and Porcine Parvovirus-Leptospira-Erysipelas (Eryseng^®^, Laboratorios Hipra, S.A., Amer, Girona, Spain). Piglets were vaccinated against Mycoplasma hyopneumoniae (Hyogen^®^, Ceva Sant´e Animale S.A., Libourne, France) at the age of 18–22 days.

## Data collection

General information about the sows, including sow identity, parity number, gestation length, body condition score, fecal score, and backfat thickness, was collected. Body condition score (on a 1–5 scale) and backfat thickness at the P2 position (measured using A-mode ultrasonography; Renco Lean-Meater^®^, MN, USA) were assessed at 109 ± 2 days of gestation. Fecal scores were evaluated individually for each sow one day prior to parturition, based on the 1–5 scale described by Oliviero et al. [11]. During parturition, various data were recorded, including farrowing duration (the interval between the expulsion of the first and last piglets), the total number of piglets born per litter, the number of piglets born alive per litter, the number of mummified fetuses, stillborn piglets per litter, and litter birth weight (kg). After parturition, the health status of the sows was monitored for 5 consecutive days. The body temperature was recorded using a digital thermometer (Omron Digital Thermometer Model MC-246, Liaoning, China). If the rectal temperature was ≥ 40 °C on the first day postpartum and/or ≥ 39.5 °C between the 2nd and 3rd day postpartum, this was defined as having fever. Vaginal discharge was scored based on appearance: 0 = clear/watery, 1 = reddish/brown, 2 = yellowish, 3 = whitish/milky [32]. Mastitis was defined by monitoring redness and swelling in the mammary gland.

## Ultrasonographic examination

**Fig. 1:**
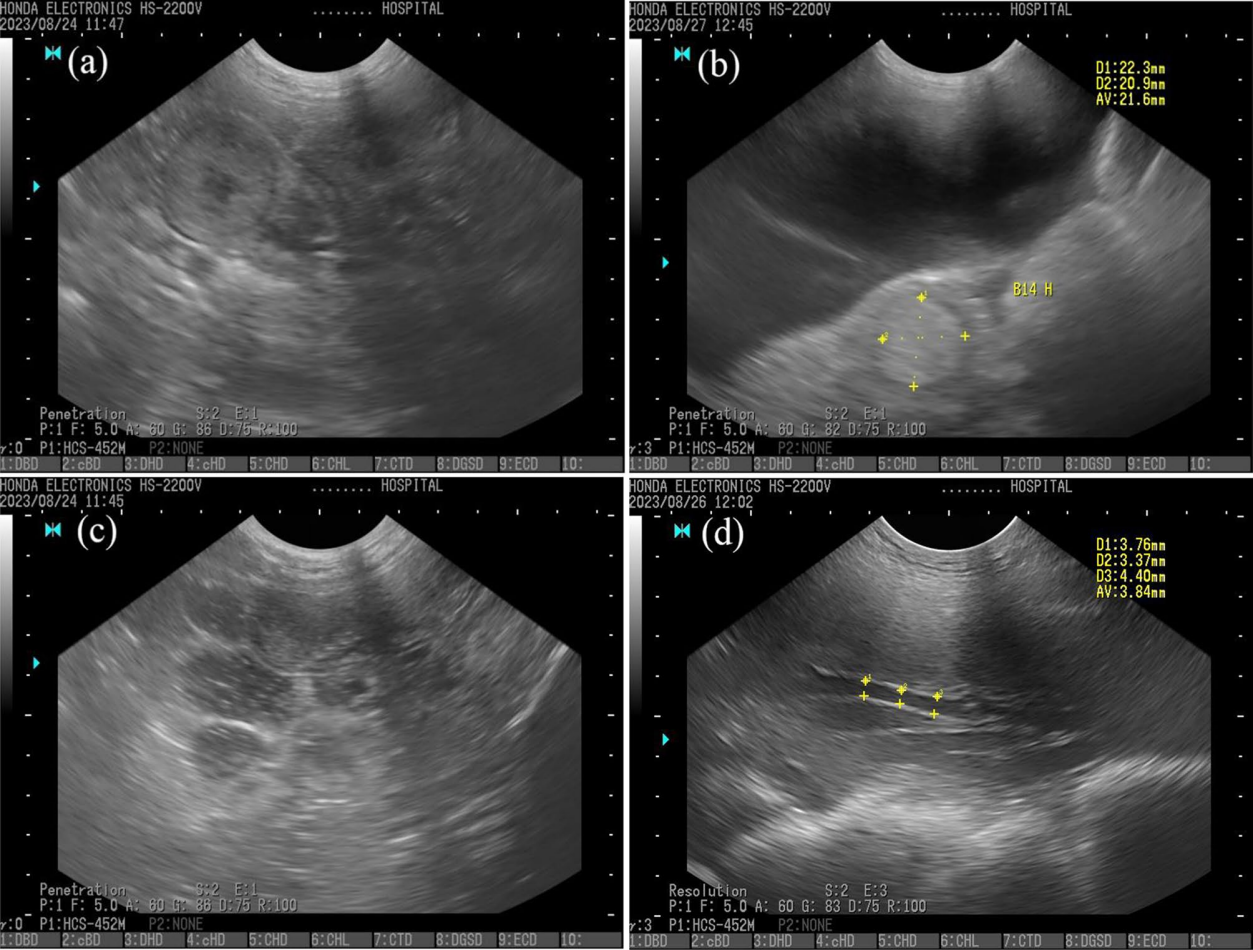
Transabdominal ultrasonographic image of (**a**) cross-sectional uterine diameter on day 2 after parturition (**b**) cross-sectional uterine diameter on day 6 after parturition, (**c**) hyperechoic intraluminal fluid accumulation in uterine horn, (**d**) uterine vessels diameter

Uterine diameter was measured using transcutaneous ultrasonographic equipment with a 5 MHz convex abdominal probe (HS-2000, Honda Electronics Co. Ltd., Aichi, Japan) on sows from days 2 to day 15 postpartum. Three cross-sectional diameters of the uterus were measured, and the average uterine diameter was calculated, displayed in millimeters (mm). Similarly, three diameters of uterine vessels in the broad ligament were measured, and an average was calculated (Fig. 1).

## Statistical analyses

All data were analyzed using SAS version 9.4 (SAS Inst. Inc., Cary, NC, USA). Descriptive statistics, including the mean, standard deviation (SD), and range of continuous variables, were calculated using the MEAN procedure in SAS. The continuous variables analyzed included parity number, body condition score, backfat thickness, gestation length, total number of piglets born per litter, number of piglets born alive per litter, number of stillborn piglets per litter, number of mummified fetuses per litter, litter birth weight, and rectal temperature after farrowing. These data are presented as mean ± SD along with the corresponding range. Additionally, reproductive parameters and farrowing traits were compared between sows with a normal farrowing duration (*n* = 10) and those with a prolonged farrowing duration (*n* = 4) using the General Linear Model (GLM) procedure in SAS. The statistical model included farrowing duration as a fixed effect. Least-square means were calculated and compared using the Least Significant Difference (LSD) test. Moreover, fecal scores (ranging from 1 to 5) were compared between the two groups using Wilcoxon’s rank sum test. The uterine diameters were analyzed using the GLM procedure of SAS. The statistical model included the effect of farrowing duration (normal vs. prolonged farrowing) as a fixed effect. The uterine diameters between the two groups were compared day by day using the LSD test. Additionally, the correlation between uterine diameter and the size of the uterine blood vessels on each day after farrowing was analyzed using Pearson’s correlation. A significance level of *P* < 0.05 was considered statistically significant.

## Results

### Descriptive statistics

All descriptive statistics on sow reproductive parameters of 14 Landrace × Yorkshire sows in a temporarily confined system under tropical climates are shown in Table 1. The average thickness of backfat in the sows was measured at 21.8 ± 4.8 mm, with values spanning from 11 to 32 mm. On average, the total number of piglets born per litter was 13.1 ± 3.5, and the number of piglets born alive per litter was 11.6 ± 3.0. The average duration of farrowing was 200.6 ± 128.3 min, with variations among sows ranging from 55 to 427 min. Of all the sows, 28.6% (4 out of 14) experienced prolonged farrowing (> 300 min). The average rectal temperature was 38.7 ± 1.0 °C, varying among sows from 37.2 to 40.8 °C, with seven out of 14 sows having fever. Twelve exhibited normal vaginal discharge, while two showed mild to moderate vaginal discharge. Among the 14 sows, 10 had a normal appetite, and four experienced a reduced appetite. Before parturition, the sows had an average body condition score of 3.7 ± 0.7, with scores ranging from 3 to 5. Table 2 presents a comparison of reproductive parameters and farrowing traits between sows with normal and prolonged farrowing durations. As shown in the table, there were no significant differences between the groups in terms of parity number, litter size, backfat thickness, or fecal score (*P* > 0.05). However, farrowing duration differed significantly between the groups (130.4 ± 18.6 vs. 376.3 ± 29.4 min, *P* < 0.001).

**Table 1:** Descriptive statistics on reproductive data of 14 landrace × Yorkshire sows in a temporarily confined system under tropical climates

Variables	Mean ± SD	Range
Parity number	2.8 ± 1.7	1–6
Body condition score (1–5)	3.7 ± 0.7	3–5
Backfat thickness (mm)	21.8 ± 4.8	11–32
Gestation length (days)	115.0 ± 0.0	115
Farrowing duration (min)	200.6 ± 128.3	55–427
Total number of piglets born per litter	13.1 ± 3.5	6–20
Number of piglets born alive per litter	11.6 ± 3.0	6–16
Number of stillborn piglets per litter	1.1 ± 1.4	0–4
Number of mummified fetuses per litter	0.4 ± 0.6	0–2
Litter birth weight (kg)	16.5 ± 4.6	10.7–26.1
Rectal temperature after farrowing (°C)		
Day 1	38.7 ± 1.0	37.2–40.8
Day 2	38.2 ± 1.1	37.2–40.1
Day 3	38.0 ± 0.8	37–39.7
Vaginal Discharge Score 0 (clear/watery)	85.7%	
Score 1 (reddish/brown)	7.1%	
Score 2 (yellowish)	0%	
Score 3 (whitish/milky)	7.1%	
Mastitis	0%	

**Table 2:** Comparison of reproductive performance and farrowing traits between sows experiencing normal (*n* = 10) and prolonged (*n* = 4) farrowing durations (least-square means ± SEM)

Variables	Normal farrowing	Prolonged farrowing	*P* value
Parity number	2.8 ± 0.5	2.8 ± 0.8	0.888
Body condition score (1–5)	3.8 ± 0.2	3.5 ± 0.4	0.507
Backfat thickness (mm)	22.5 ± 1.5	20.1 ± 2.4	0.418
Gestation length (days)	115.0 ± 0.0	115.0 ± 0.0	1.000
Farrowing duration (min)	130.4 ± 18.6	376.3 ± 29.4	< 0.001
Total number of piglets born per litter	13.5 ± 1.1	12.3 ± 1.8	0.567
Number of piglets born alive per litter	12.2 ± 0.9	10.3 ± 1.5	0.289
Number of stillborn piglets per litter	0.9 ± 0.5	1.5 ± 0.7	0.503
Number of mummified fetuses per litter	0.4 ± 0.2	0.5 ± 0.3	0.805
Litter birth weight (kg)	16.4 ± 1.5	16.8 ± 2.4	0.916
Fecal score (1–5)	2.80 ± 0.79	3.00 ± 0	0.773

## Uterine diameters

**Fig. 2:**
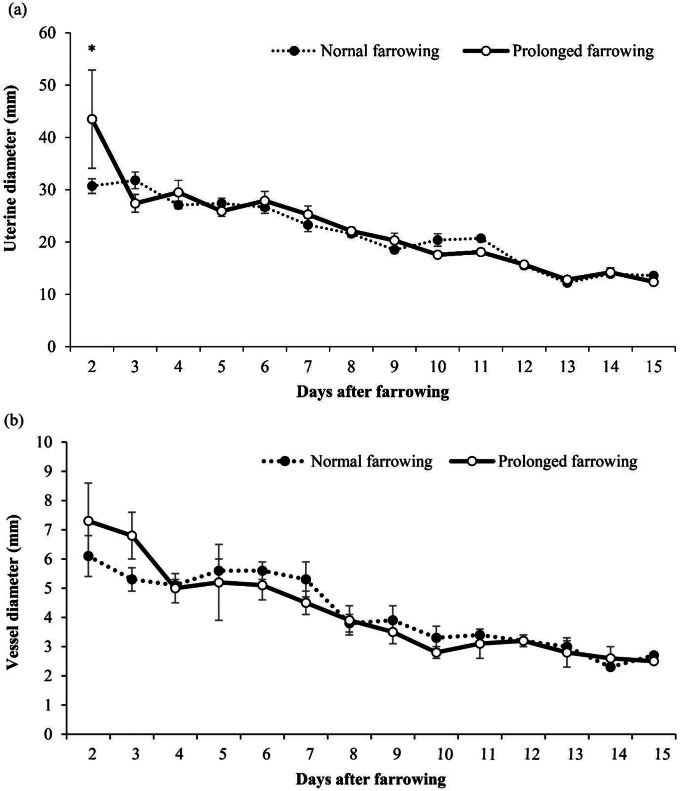
(**a**) Uterine diameter measurements from days 2 to 15 postpartum in sows in a temporarily confined system. *Indicate significant differences (*P* < 0.05) (**b**) Uterine vessel diameter measurements from days 2 to 15 postpartum in sows in a temporarily confined system (means ± SEM)

The measurement of uterine diameters from day 2 to day 15 postpartum is shown in Fig. 2a. The uterine diameters of sows in both groups decreased from day 2 to day 15 postpartum. In sows with prolonged farrowing duration, the diameter decreased from 43.5 ± 9.4 mm to 12.4 ± 0.5 mm, while in sows with normal farrowing duration, it decreased from 30.7 ± 1.4 mm to 13.6 ± 0.4 mm. At 2 days postpartum, a significant difference (*P* = 0.042) in uterine diameter was observed between sows with prolonged farrowing (43.5 ± 9.4 mm) and those with normal farrowing (30.7 ± 1.4 mm).

### Uterine vessels diameters

**Fig. 3:**
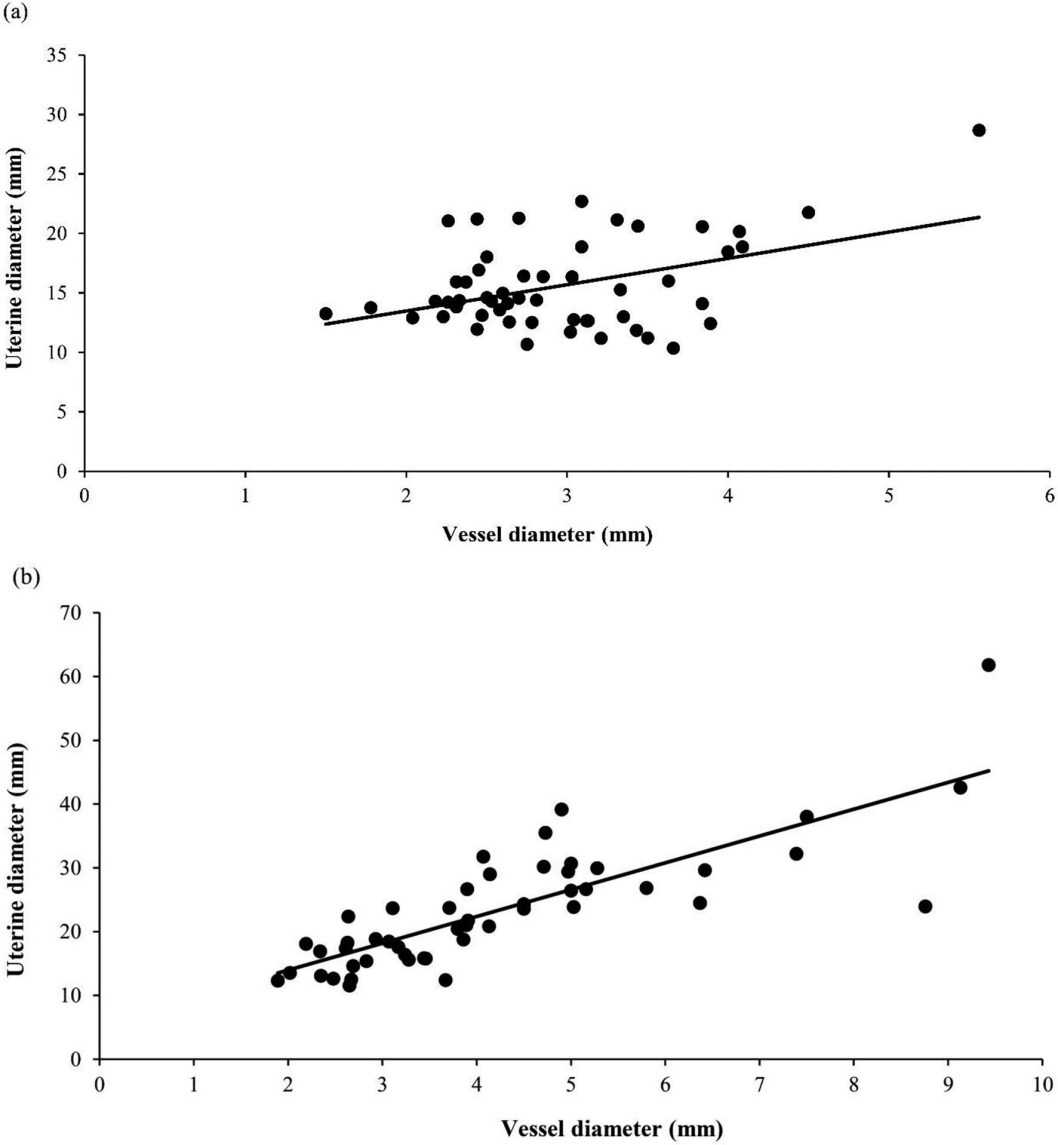
Correlation between uterine diameter and vessel diameter in sows with normal farrowing duration (*r* = 0.705, *n* = 10, *P* < 0.001) (**a**), and in sows with prolonged farrowing duration (*r* = 0.749, *n* = 4, *P* < 0.001) (**b**)

The uterine vessel diameters from day 2 to day 15 postpartum are shown in Fig. 2b. In sows with prolonged farrowing duration, the diameter decreased from 7.3 ± 1.3 mm to 2.5 ± 0.1 mm from day 2 to day 15 postpartum (*P* < 0.001), while in sows with normal farrowing duration, it decreased from 6.1 ± 0.7 mm to 2.7 ± 0.1 mm over the same period (*P* < 0.001). Throughout the study period, a significant positive correlation was found between uterine diameter and uterine blood vessel size in sows with both normal (*r* = 0.705, *P* < 0.001; Fig. 3a) and prolonged farrowing durations (*r* = 0.749, *P* < 0.001; Fig. 3b). However, at 2 days postpartum, no significant difference in vessel diameter was observed between sows with prolonged farrowing (7.3 ± 1.3 mm) and those with normal farrowing (6.1 ± 0.7 mm, *P* = 0.397).

## Discussion

This study aimed to assess the relationship between uterine involution and farrowing duration. The study revealed that uterine diameters of sows in both groups decreased from day 2 to day 15 postpartum. Sows with a prolonged farrowing duration exhibited a larger initial uterine diameter on the second day postpartum compared to those with a normal farrowing duration. Subsequently, the uterine diameter progressively decreased in both groups. A significant difference between the two groups was observed only at 2 days postpartum, with no significant differences detected on the following days. This finding agrees with the previous study [24], which demonstrated that crated sows with prolonged farrowing duration had a larger uterus at the beginning of the postpartum period compared to sows that had a normal farrowing duration. It is well established that farrowing duration in sows is influenced by several factors, including parity number, body condition score, backfat thickness prior to parturition, and constipation [8, 11, 15]. However, the effect of prolonged farrowing duration on the rate of uterine involution in sows under tropical conditions has not been clearly demonstrated. In the present study, the parity number, body condition score, and backfat thickness were balanced between sows with normal and prolonged farrowing durations, allowing for an unbiased comparison of the impact of prolonged parturition on uterine involution.

A novel finding of the present study is that the diameter of uterine vessels in sows with prolonged farrowing duration decreased from 7.3 mm to 2.5 mm (a 65.8% reduction) within 15 days postpartum, while in sows with normal farrowing duration, it decreased from 6.1 mm to 2.7 mm (a 55.7% reduction) over the same period. Although the uterine vessel diameter was numerically larger in sows with prolonged farrowing duration, the difference was not statistically significant compared to those with normal farrowing duration. This suggests that sows experiencing prolonged farrowing may initially exhibit a slightly larger uterine vessel diameter than those with normal farrowing. However, the diameter progressively declined in both groups, following a similar pattern to the reduction in uterine diameter. Previous studies in human medicine using ultrasonography to measure uterine vessels demonstrated that the uterine vessel diameter was larger in pregnant women compared to non-pregnant women [31]. In humans, uterine involution is characterized not only by a gradual reduction in uterine size and cavity dimensions but also by dynamic changes in uterine artery blood flow indices, as measured by Doppler ultrasound [33–35]. This process is primarily driven by a marked decline in uterine vascularity, especially during the first 2 weeks postpartum [33]. Additionally, a study on sacrificed sows investigated structural changes in the uterine artery in the first trimester of pregnancy and found that the uterine artery underwent physical-mechanical changes to increase blood flow to maintain pregnancy [36]. Interestingly, this study is the first to show that uterine vessels are initially larger during the early postpartum days and progressively decrease in size until 15 days after parturition. This suggests that accurately measuring the diameter of uterine vessels in postpartum sows could serve as an additional method for indicating physiological changes in the uterus during the postpartum period.

In the present study, the mean farrowing duration was 200.6 min, with 28.6% of sows (4 out of 14) experiencing prolonged farrowing (> 300 min). This aligns with a previous study in Thailand using a free-farrowing system, which reported a mean farrowing duration of 221 min, with 21.7% experiencing prolonged farrowing [8]. Risk factors significantly increasing the farrowing duration of sows in the free-farrowing system included the total number of piglets born per litter, number of stillborn piglets, number of mummified fetuses, number of placental parts, obstetrical intervention, piglet birth weight, and litter birth weight [8, 19, 20]. Moreover, sows with a parity number of ≥ 5 had a longer farrowing duration than primiparous sows and sows with parity numbers of 2 to 4 [8]. Interestingly, in multiparous sows, a positive correlation was observed between the length of the farrowing process and the highest temperature, as well as the peak temperature-humidity index, during the 7 days leading up to farrowing [8]. This information suggests that sows exposed to moderate to severe heat stress may experience various physiological alterations in uterine involution compared to those in temperate zones, highlighting the need for further research to comprehensively understand these changes under different conditions. In addition to the aforementioned factors, there are other known influencers of farrowing duration such as constipation, body condition score, genetics, gestation length, and housing [28, 37]. In the present study, the average body condition score of pre-partum sows was 3.7, with an average backfat thickness of 21.8 mm, both of which were considered relatively high [38]. These factors may have partially influenced farrowing performance and subsequently contributed to excessive backfat loss during lactation due to reduced appetite [38]. Tummaruk [38] demonstrated that the proportion of sows losing more than 10% of backfat during lactation was higher among those with pre-farrowing backfat thickness greater than 25.0 mm compared to those with 15.0 to 20.0 mm. This could represent another potential factor compromising the uterine involution process in sows. Studies regarding housing demonstrate that sows in a free-farrowing system tend to have shorter farrowing durations than those in crate systems, which is attributed to higher oxytocin levels [37]. Sows in free-farrowing systems also show smaller uterine diameters compared to those in crate housing [22]. This difference is attributed to the ability of sows in free-farrowing systems to express nesting behavior, which affects hormone regulation and decreases stress levels during the farrowing process [22, 25, 26]. Currently, ultrasonography is used in pigs for more than just pregnancy examinations. Studying uterine involution helps veterinarians to detect uterine disorders and determine the optimal solutions for treating sows during the postpartum period [23, 25, 26]. Further studies are needed to identify the risk factors and optimal management solutions to reduce the incidence of poor uterine involution in sows during the postpartum period. For example, under tropical climates, sows are often exposed to temperatures exceeding the optimal range for lactating sows (12–22 °C) prior to parturition [39, 40]. Therefore, maintaining temperature and humidity levels within the optimal range before farrowing is critically important for swine production in tropical regions. Additionally, since body condition score and backfat thickness before farrowing are key risk factors for negative energy balance and excessive backfat loss during lactation [38], further studies investigating the optimal backfat thickness for modern genetic lines of sows would be highly valuable. The ultimate goal is to improve the health status of postpartum sows, particularly those that are hyperprolific and regularly exposed to moderate to severe heat stress before parturition.

A recent study conducted under tropical conditions with a large sample size reported that approximately 21% of sows experienced prolonged farrowing durations (> 300 min) [40]. Factors significantly associated with prolonged farrowing included litter size, number of stillborn piglets, sow parity, timing of farrowing onset, month of farrowing, and climatic parameters [40]. For example, sows that began farrowing during working hours had significantly longer farrowing durations, averaging 29.8 min more than those that farrowed during non-working hours [40]. Moreover, under tropical climates, farrowing duration was highly sensitive to environmental conditions: each 1 °C increase in temperature during the 7 days preceding parturition was associated with a 4.3-min increase in farrowing duration, while a 10% rise in relative humidity resulted in a 21-min increase [40]. These findings emphasize the considerable influence of climatic factors on farrowing performance. However, such effects could not be demonstrated in the present dataset, as all sows farrowed within the same week and parity numbers were balanced between groups.

The limitation of the present study stems from the fact that it was conducted under field conditions, where the number of sows with prolonged farrowing duration could not be fully controlled. The primary objective of our study was to compare uterine and vascular dimensions in sows experiencing normal versus prolonged parturition (defined as > 300 min). According to Akkhaphan et al. [40], 21.1% of sows in a given herd experienced prolonged parturition over the course of a year. In our study, 4 out of 14 sows (28.5%) had farrowing durations exceeding 300 min, which aligns with the earlier report and reflects the typical proportion of sows with prolonged parturition in each group. To control for environmental variables, all sows in both the normal and prolonged parturition groups were selected from the same weekly farrowing batch. This approach also allowed us to monitor the same animals from farrowing through to 15 days postpartum. Additionally, all ultrasound examinations were conducted by a single technician to minimize inter-observer variability. Consequently, only 14 sows that farrowed within the same week and were scanned by the same technician were included in this pilot study. Furthermore, this study did not clearly distinguish between uterine arteries and veins, despite potential anatomical differences in their size and function that could have influenced the results.

## Conclusions

In summary, sows in the temporarily confined system under tropical climates with prolonged farrowing exhibited a notable increase in uterine size during the initial two days postpartum. Consequently, it is essential to enhance awareness and implement targeted care for sows that experienced prolonged farrowing. Furthermore, this study represents the first investigation into the diameter of uterine vessels in the puerperium of sows and its correlation with the uterine diameter. Further research is needed to assess whether the size of uterine vessels can serve as an indicator of uterine disorders after farrowing.

## Data Availability

No datasets were generated or analysed during the current study.
